# Decreased Diastolic Ventricular Kinetic Energy in Young Patients with Fontan Circulation Demonstrated by Four-Dimensional Cardiac Magnetic Resonance Imaging

**DOI:** 10.1007/s00246-016-1565-6

**Published:** 2017-02-10

**Authors:** Pia Sjöberg, Einar Heiberg, Pär Wingren, Jens Ramgren Johansson, Torsten Malm, Håkan Arheden, Petru Liuba, Marcus Carlsson

**Affiliations:** 1 Department of Clinical Sciences Lund, Clinical Physiology, Skane University Hospital, Lund University, Lund, Sweden; 2 Department of Clinical Sciences Lund, Pediatric Heart Center, Skane University Hospital, Lund University, Lund, Sweden; 3 Department of Biomedical Engineering, Faculty of Engineering, Lund University, Lund, Sweden; 4 Center for Mathematics, Faculty of Engineering, Lund University, Lund, Sweden; 5 Department of Medical Imaging and Physiology, Skane University Hospital, Lund, Sweden

**Keywords:** Fontan, Congenital heart disease, Kinetic energy, CMR, MRI, Magnetic resonance imaging

## Abstract

Four-dimensional (4D) flow magnetic resonance imaging (MRI) enables quantification of kinetic energy (KE) in intraventricular blood flow. This provides a novel way to understand the cardiovascular physiology of the Fontan circulation. In this study, we aimed to quantify the KE in functional single ventricles. 4D flow MRI was acquired in eleven patients with Fontan circulation (median age 12 years, range 3–29) and eight healthy volunteers (median age 26 years, range 23–36). Follow-up MRI after surgical or percutaneous intervention was performed in 3 patients. Intraventricular KE was calculated throughout the cardiac cycle and indexed to stroke volume (SV). The systolic/diastolic ratio of KE in Fontan patients was similar to the ratio of the controls’ left ventricle (LV) or right ventricle (RV) depending on the patients’ ventricular morphology (Cohen´s *κ* = 1.0). Peak systolic KE/SV did not differ in patients compared to the LV in controls (0.016 ± 0.006 mJ/ml vs 0.020 ± 0.004 mJ/ml, *p* = 0.09). Peak diastolic KE/SV in Fontan patients was lower than in the LV of the control group (0.028 ± 0.010 mJ/ml vs 0.057 ± 0.011 mJ/ml, *p* < 0.0001). The KE during diastole showed a plateau in patients with aortopulmonary collaterals. This is to our knowledge the first study that quantifies the intraventricular KE of Fontan patients. KE is dependent on the morphology of the ventricle, and diastolic KE indexed to SV in patients is decreased compared to controls. The lower KE in Fontan patients may be a result of impaired ventricular filling.

**Electronic supplementary material:**

The online version of this article (doi:10.1007/s00246-016-1565-6) contains supplementary material, which is available to authorized users.

## Introduction

The Fontan operation is the procedure of choice used to palliate patients with a congenital heart defect not suited for biventricular repair [1]. One functional ventricle pumps the blood through the systemic circulation and the blood returns passively to the pulmonary circulation through the caval veins.

The Fontan circulation is dependent on an elevated systemic venous pressure, low pulmonary vascular resistance, and low left atrial pressure [2, 3]. The patients are also dependent on a systemic ventricle with good systolic function, but since the primary limitation of the Fontan circulation is the filling of the systemic ventricle, a good diastolic function is mandatory for preserving an adequate cardiac output [4].

Studies show decrease in survival beyond 15–20 years after surgery in Fontan patients [5, 6] and the number of additional interventions after the Fontan procedure is high [7]. This is mainly driven by the prognosis of the Fontan patients who develop complications due to longstanding low cardiac output [8]. Increased understanding of the Fontan circulation physiology is therefore central to be able to prevent and treat possible complications.

The ability of the ventricle to deliver cardiac output to the body is facilitated by the looped shape anatomy of a morphological left ventricle that helps to conserve the momentum of blood [9] through a ventricular inflow vortex [10, 11]. This means that the momentum of blood is preserved during diastole in a vortex and directed toward the outflow tract [11]. Four-dimensional (4D) phase-contrast cardiac magnetic resonance imaging (MRI) measures velocities of the blood in three directions over time and has been used to improve the accuracy in flow measurement over the atrioventricular valve in patients with Fontan circulation [12]. Furthermore, 4D flow enables quantification of caval flow distribution to the pulmonary artery [13]. 4D flow MRI can also quantify the kinetic energy (KE) of the heart chambers [14–19] and hence provide fundamental insights into these aspects of heart physiology. Studies in adult patients with heart failure and patients with mitral regurgitation have shown alterations in KE [20–22] and recently Jeong et al. used this method to quantify right ventricular KE in Tetralogy of Fallot patients [23]. Knowledge of KE in single ventricles and Fontan circulation may improve our understanding of the hemodynamic reasons for impaired function in these patients, but to our knowledge there are no published studies to date on ventricular KE data in these patients.

Therefore, the aim of this study was to noninvasively quantify the kinetic energy during the entire cardiac cycle of the single ventricle in Fontan patients using four-dimensional phase-contrast magnetic resonance imaging (4D PC-MRI) to better understand the circulation and the pathophysiology of complications.

## Materials and Methods

### Study Design

Patients with Fontan circulation referred to Skane University Hospital for a cardiovascular magnetic resonance (CMR) examination were prospectively included. The inclusion time was from November 2013 until September 2014. In addition to routine clinical examination, 4D flow was acquired. Eight healthy volunteers (2 females, median age 26 years, range 23–36) were used as controls. Healthy controls were recruited by advertising at the local institution and had to have blood pressure <140/90, normal ECG, no cardiovascular medication, and no medical history of cardiovascular or other systemic disease to be included. The Fontan KE was compared on a group level to the LV KE of controls. The reason for comparing with the LV was that even if there is an RV morphology in a Fontan patient, the systemic ventricle is pumping with the requirements closer to an LV rather than an RV.

The principles of the Helsinki declaration were followed and the study was approved by the Regional Ethical Review Board, Lund, Sweden. Written informed consent was obtained from all subjects or their parents if <18 years of age.

### Magnetic Resonance Imaging

4D PC-MRI flow acquisitions and cine images were acquired as previously described and validated in vivo and in vitro [24, 25]. In short, images were acquired using retrospective ECG triggering, with a 1.5 or 3 T Philips Achieva MRI. A volume covering the entire heart and central great vessels was acquired using a 4D phase-contrast velocity mapping turbo-field-echo sequence. Typical imaging parameters were TR/TE/flip angle: 6.2 ms/3.6 ms/8° and velocity encoding 100 cm/s. Number of time phases acquired was dependent on heart rate and set to the maximum with a segmentation factor of 2. The acquired temporal resolution varied from 50 to 55 ms, i.e., 14–22 phases acquired and thereafter reconstructed to 40 time phases. Voxel size was 2 × 2 × 2 mm in children and 3 × 3 × 3 mm in adults. Acquired matrix size was typically 80 × 80 × 35. Images were acquired during free breathing without navigator triggering as earlier described and validated [26].

A steady-state free precession sequence (TR/TE/flip angle: 2.9 ms/1.5 ms /60°, slice thickness 8 mm, in-plane resolution 1.2 × 1.2 mm) was used to collect cine images covering the entire heart. Flow measurements with a phase-contrast velocity mapping fast field echo sequence (TR/TE/flip angle: 10 ms/6.5 ms/15° in-plane resolution 1.2 × 1.2 mm) during free breathing as earlier described and validated were performed in the ascending aorta to measure the effective stroke volume (SV) and cardiac index (CI) [27].

Gadolinium contrast is not required for the 4D flow acquisitions and was not given as part of the study protocol but was administered in 2/11 patients due to clinical questions of myocardial fibrosis in one case and MR angiography in one case.

### Image Analysis

Images were analyzed with the Segment software (http://segment.heiberg.se), using an in-house developed module for 4D PC-MRI analysis of KE [16]. A first-order polynomial fit to stationary tissue was used to compensate for eddy currents. Maxwell effects due to concomitant gradients were compensated for by the scanner. Velocity aliasing was corrected using phase unwrapping. The net three-dimensional velocity vector for each voxel was computed.

Endocardial contours of the functional single ventricle and the contours of the superior vena cava (SVC), the tunnel or conduit from the inferior vena cava (IVC), and the pulmonary artery (PA) branches were manually traced in all phases of the cine images. The segmentation was imported to the reconstructed 4D flow dataset with manual correction when needed. KE was calculated within the resulting delineation of the ventricle throughout the cardiac cycle in the 4D flow images. The KE of a voxel was calculated as KE=½*mv* ^2^, where *m* is the mass of the blood in the voxel and *v* is the velocity of the voxel. The mass of the voxel was calculated as the volume multiplied by the density of the blood, 1.05 g/cm^3^ [28]. The KE of each voxel within the delineation of the ventricle or vessel was summed to get the KE of the entire ventricle or vessel part in each phase of the cardiac cycle. To be able to compare patients with different age and body size, KE was indexed to stroke volume (SV) or the volume of the vessel part.

The location of the KE in the ventricle was performed by exporting velocity data and anatomical images from Segment to the open-source 4D flow software FourFlow (http://fourflow.heiberg.se) enabling anatomical correlation of KE. Peak systolic and diastolic velocities were quantified in the outflow and inflow of the ventricles, and correlated to systolic and diastolic peak KE, respectively. The electronic supplement file Movie 1 shows step by step how KE was analyzed using 4D flow MRI.

### Clinical Evaluation

Patients’ clinical status was evaluated by their treating pediatric cardiologist. Aortopulmonary collaterals (APCs) were evaluated during catheterization using fluoroscopic angiography of the thoracic aorta and the degree of collateral flow from MRI. APC blood flow was calculated using MRI by subtracting the flow volume in the caval veins from the aortic flow measured by 2D flow. The difference between pulmonary and caval venous flows was used for APC quantification in three patients where aortic flow measurements were not available [29]. Seven of the 11 patients underwent catheterization as part of the clinical evaluation. Hemodynamically significant APCs were defined as enlarged APC with saturation step-up in the pulmonary artery branches in combination with elevated end-diastolic pressure (measured by catheterization) and >25% APC flow contribution to SV. Complications were defined as the need of intervention in the Fontan circulation.

### Statistical Analysis

Statistical analysis was performed using GraphPad (v6.04, La Jolla, CA; USA). Ventricular KE is presented as means ± SD. Differences in KE between Fontan patients and healthy volunteer groups were assessed using the Mann–Whitney test.

Since the number of patients in each group was small, statistical comparisons were not performed for differences between subgroups.

Cohen´s kappa was analyzed for the relation of KE pattern and ventricular morphology.

## Results

Eleven patients with Fontan circulation underwent 4D PC-MRI. One example of intracardiac and cavopulmonary blood flow is shown in Movie 2 in electronic supplements. The patients’ characteristics are shown in Table 1.

**Table 1:** Patient’s characteristics

Subject	Age at time of CMR (y)	Gender	BSA (m2)	Ventricular morphology	Type of Fontan	Diagnosis	APC	Complications	NYHA
1	3	M	0.56	Left	Extracardiac	Unbalanced AVSD, small LV, PA, MAPCA, right isomerism, bilateral SVC, dextrocardia. Fenestrated*	*	PLE	NYHA II
2	7	M	0.82	Left	Extracardiac	HLHS	Yes	Restrictive ASD	NYHA III
3	4	M	0.68	Right	Extracardiac	HLHS, TAPVD, bilateral SVC	Yes	PLE	NYHA IV
4	12	F	1.26	Right	Extracardiac	Hypoplastic right PA, left isomerism, interrupted IVC, dextrocardia	No	Pulmonary arteriovenous fistulas	NYHA IV
5	4	M	0.72	Right	Extracardiac	Hypoplastic aortic arch, CoA, VSD, DORV	Yes	LPA stenosis	NYHA II
6	15	M	1.60	Right	Extracardiac	DORV Taussig Bing, unbalanced AVSD, PS, TAPVD, right isomerism	Yes	APC	NYHA II
7	17	M	1.72	Right	Lateral tunnel	Single ventricle, DORV, TGA, dextrocardia	No	No	NYHA I
8	14	F	1.37	Right	Extracardiac	Single ventricle, PS, TGA, right isomerism, bilateral SVC, TAPVD	Yes	No	NYHA I
9	13	F	1.36	Left	Extracardiac	TGA, multiple VSD, hypoplastic pulm arteries	Yes	No	NYHA I
10	29	M	1.72	Left	Right atrium to PA	Tricuspid atresia	No	No	NYHA I
11	4	M	0.72	Left with long outflow tract	Extracardiac	DILV, TGA, PS, left pulm artery stenosis	No	No	NYHA II

*CMR* cardiac magnetic resonance; *BSA* body surface area; *APC* aortopulmonary collaterals; *NYHA* New York Heart Association Functional Classification; *AVSD* atrioventricular septal defect; *LV* left ventricle; *MAPCA* major aortopulmonary collateral arteries; *SVC* superior vena cava; *HLHS* hypoplastic left heart syndrome; *TAPVD* total anomalous pulmonary venous drainage; *IVC* inferior vena cava; *CoA* coarctation of aorta; *VSD* ventricular septal defect; *DORV* double outlet right ventricle; *PS* pulmonary stenosis; *TGA* transposition of the great arteries; *DILV* double inlet left ventricle; *PLE* protein-losing enteropathy; *ASD* atrial septal defect; *LPA* left pulmonary artery

*Fenestrated extracardiac tunnel which makes the assessment of APC difficult

Five clinically stable patients (2 female, median age 14, range 4–29) and 6 patients (1 female, median age 5, range 3–15) who had complications were included in the study. Six patients had complications with need of intervention for pulmonary branch stenosis in four cases, restrictive atrial communication in one case, and severe APC in one case. Three patients underwent a follow-up MRI after surgical or percutaneous intervention. The interventions are described in Table 2. Five patients were below 10 years of age and these examinations were performed under general anesthesia.

**Table 2:** Characteristics and type of intervention in three patients who underwent cardiac magnetic resonance before and after intervention

Subject	Diagnosis	Type of Fontan	Reason for intervention	Intervention
4	Hypoplastic right pulmonary artery, left isomerism, interrupted IVC, dextrocardia	Extracardiac	Pulmonary arteriovenous fistula. All liver drainage to one lung	Extracardiac conduit was converted to a Y-graft from the inferior vena cava to both pulmonary branches separately
5	Hypoplastic aortic arch, CoA, VSD, DORV	Extracardiac	Stenosis of left pulmonary artery	Dilatation and stenting of left pulmonary artery
6	DORV Taussig Bing, unbalanced AVSD, PS, TAPVD, right isomerism	Extracardiac	APC	Coiling of collaterals

*IVC* inferior vena cava; *CoA* coarctation of aorta; *VSD* ventricular septal defect; *DORV* Double outlet right ventricle; *AVSD* atrioventricular septal defect; *PS* pulmonary stenosis; *TAPVD* total anomalous pulmonary venous drainage; *APC* aortopulmonary collaterals

### KE Pattern and Location in the Ventricle

**Fig. 1:**
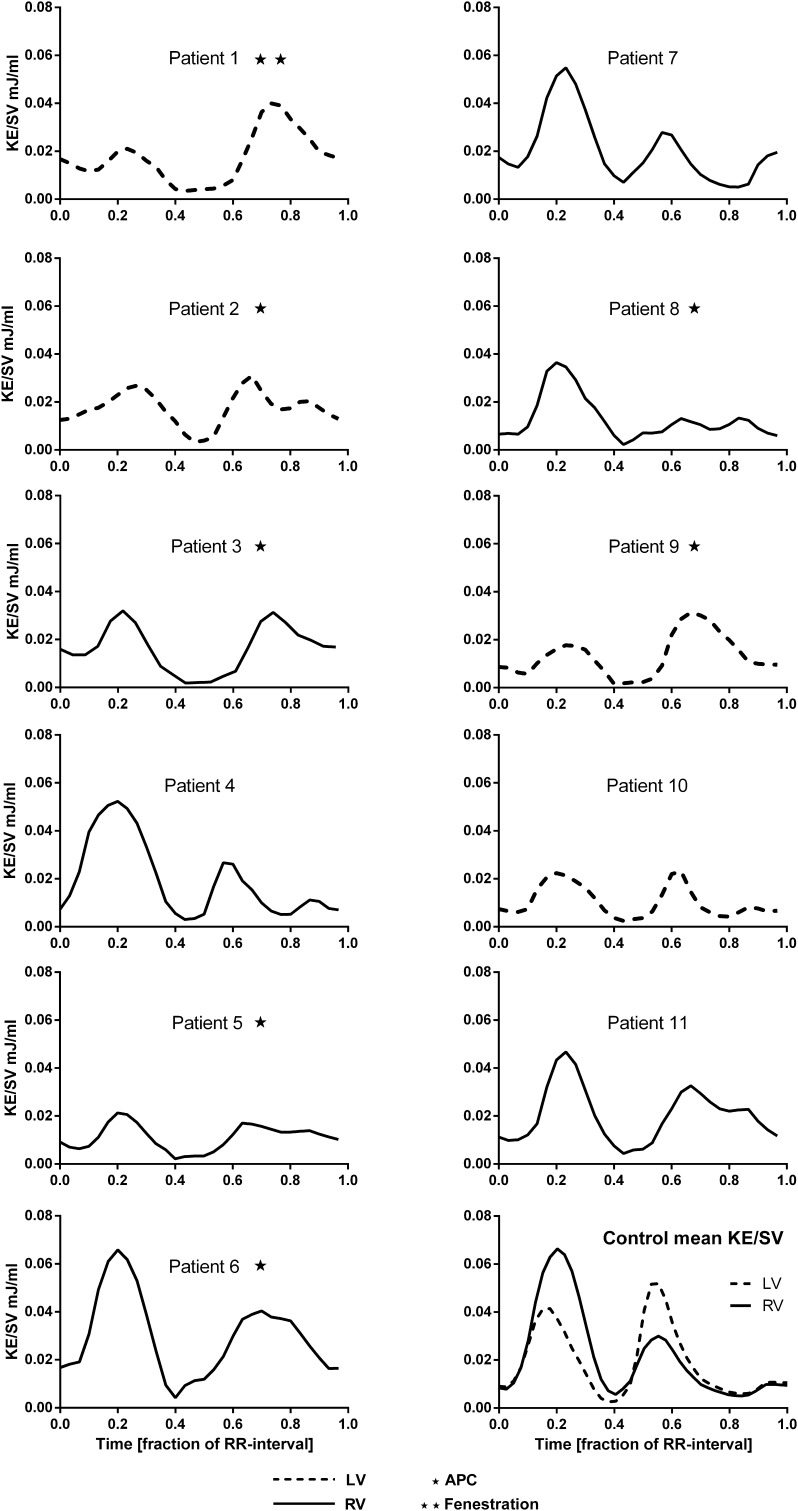
Kinetic energy in the ventricle during the cardiac cycle. Right ventricular (RV) morphology is shown in *solid line*. Left ventricular (LV) morphology is shown in *broken line*. The *left column* shows Fontan patients with complications. The *right column* shows patients with Fontan circulation without complications and a graph showing RV and LV of the control group. Patients with APC (aortopulmonary collaterals) are marked with a *star*. Patient 1 had a fenestrated extracardiac conduit which makes the assessment of APC difficult

**Fig. 2:**
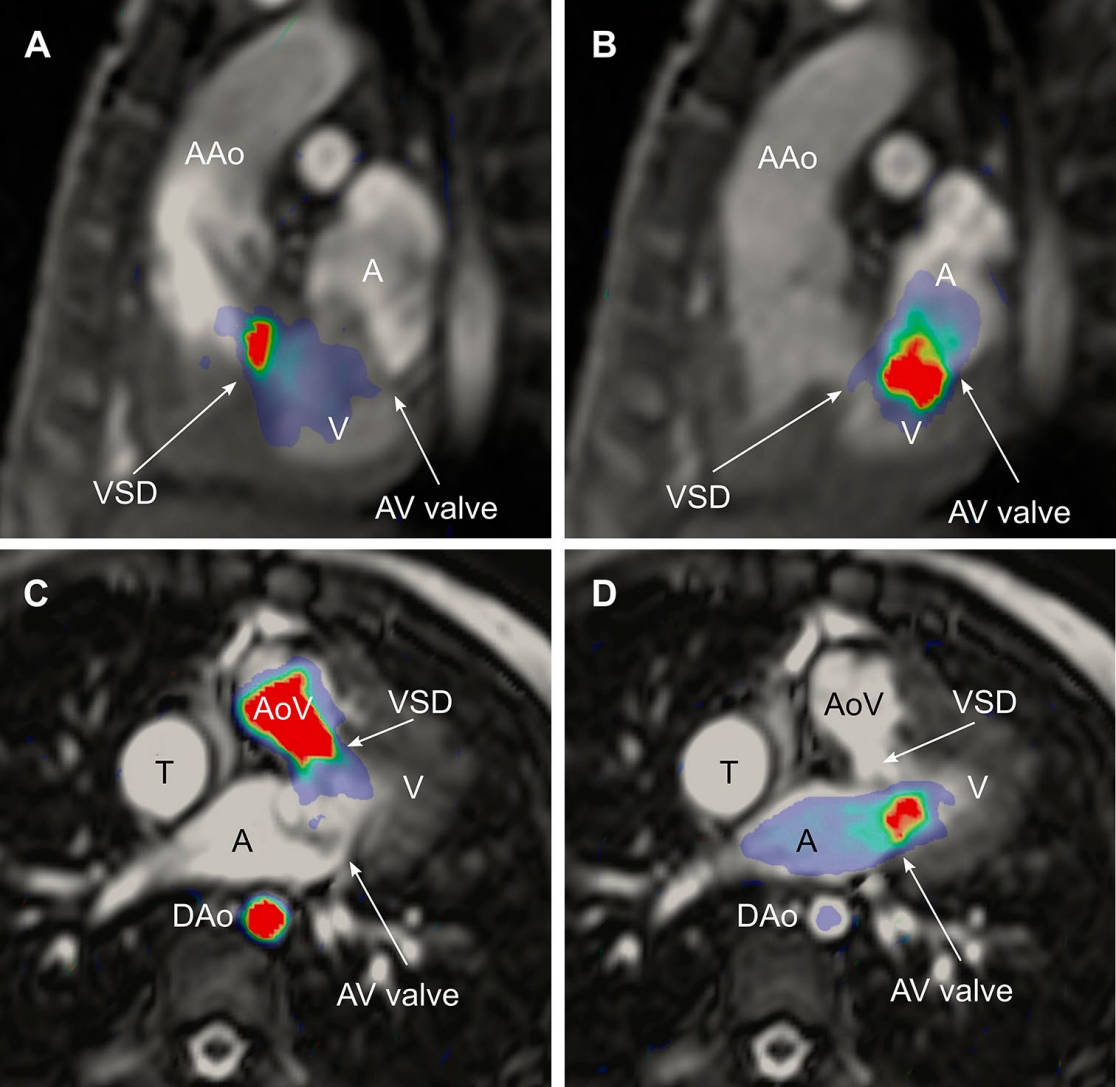
Ventricular kinetic energy (KE) in a Fontan patient superimposed on CMR images to visualize the anatomical location of KE. The *upper panel* shows an oblique sagittal view in systole (**a**) and diastole (**b**). Systolic peak KE can be seen in a ventricular septal defect leading the blood to the aorta. Diastolic peak KE is located from the atrioventricular valve into the ventricle. The *lower panel* visualizes KE in an oblique transversal view in the same patient in systole (**c**) and diastole (**d**). *AAo* ascending aorta; *VSD* ventricular septal defect; *V* ventricle; *A* atrium; *AV valve* atrioventricular valve; *AoV* aortic valve; *DAo* descending aorta; *T* lateral tunnel

In all Fontan patients, two KE peaks were seen, one in systole and one in diastole. The KE throughout the cardiac cycle or the KE patterns is shown in Fig. 1. Three patients also had a late diastolic peak, corresponding to the atrial contraction, while the peaks during early filling phase and atrial contraction were completely or partially fused in the remaining subjects. Patients with a left ventricular morphology with a short outflow tract (*n* = 4) had a lower systolic KE peak and a relatively higher diastolic peak (systole/diastole <1). Patients with right ventricular morphology and a long outflow tract (*n* = 6) had a higher systolic than diastolic peak (systole/diastole >1). One patient, number 11, had a morphological left ventricle (LV) where the blood had to pass through a VSD via small RV to the aorta. A high diastolic peak, but even higher systolic peak, was seen in this patient (systole/diastole >1). Visualization showed that the KE in systole was mainly located in the outflow tract and during diastole from the mitral valve into the ventricle. KE in one patient is visualized as an example in Fig. 2. Cohen´s kappa for ventricular morphology and KE pattern was 1.0. Six patients had hemodynamically significant aortopulmonary collaterals (APCs) with a mean flow of 35% ± 9. Patients with APC had a diastolic plateau of KE but this was only seen in one of the patients (Patient 11) without significant APC (Fig. 1). Patients without significant APC had a mean APC flow of 17% ± 9. Patient 11 had an APC flow of 6%.

### KE Values

**Fig. 3:**
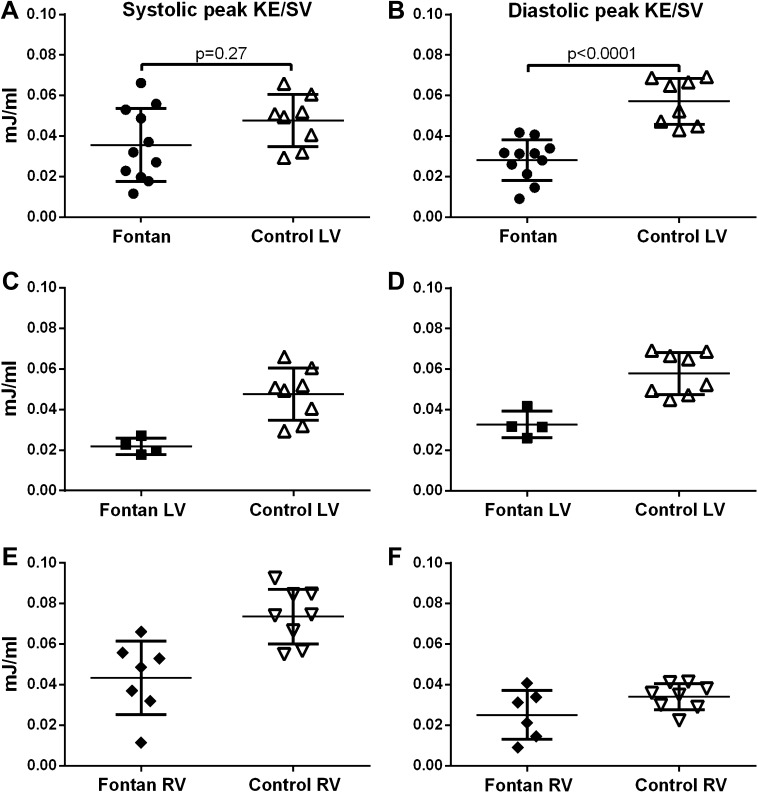
Kinetic energy (KE) indexed to stroke volume. The *left column* shows peak KE during systole and the *right column* peak KE during diastole. **a** and **b** shows all Fontan patients and the left ventricle in the control group. **c** and **d** shows Fontan patients with LV morphology and the left ventricle in the control group. **e** and **f** shows Fontan patients with RV morphology and the right ventricle in the control group. *Error bars* show mean ± SD

No significant difference was seen in systolic peak KE/SV in the Fontan group (0.036 ± 0.018 mJ/ml, *n* = 11) compared to the LV in controls (0.048 ± 0.012 mJ/ml, *n* = 8, *p* = 0.27, Fig. 3a). The diastolic peak KE/SV in Fontan patients (0.028 ± 0.010 mJ/ml) was lower than in the LV of the control group (0.057 ± 0.011 mJ/ml, p < 0.0001, Fig. 3b).

Peak KE/SV in systole in stable patients was 0.036 ± 0.016 mJ/ml and in patients with complications 0.035 ± 0.021 mJ/ml. Peak KE/SV in diastole for stable vs patients with complications were 0.027 ± 0.007 mJ/ml and 0.029 ± 0.012 mJ/ml.

Table 3 and Fig. 3c–f show the KE values for Fontan patients and controls divided into subgroups based on ventricular morphology. Statistical comparisons could not be made due to the limited sample size when subdividing the group, but Fontan patients tended to show lower values compared to healthy volunteers.

**Table 3:** Kinetic energy indexed to stroke volume, body surface area, and cardiac index in Fontan patients compared to healthy controls, divided into type based on ventricular morphology

Ventricular morphology		Fontan patients	Controls
Left ventricle Fontan, *n* = 4 Control, *n* = 8	EDVI, ml/m^2^	102 ± 42	101 ± 11
	ESVI, ml/m^2^	60 ± 29	43 ± 8
	EF, %	43 ± 6	57 ± 5
	CO, l/min	3.7 ± 2.4	6.5 ± 1.2
	CI, l/min/m^2^	4.0 ± 1.5	3.3 ± 0.4
	Peak systolic KE/SV, mJ/ml	0.022 ± 0.004	0.048 ± 0.012
	Peak diastolic KE/SV, mJ/ml	0.033 ± 0.007	0.058 ± 0.010
	Peak systolic KE/BSA, mJ/m^2^	0.56 ± 0.20	2.57 ± 0.60
	Peak diastolic KE/BSA, mJ/m^2^	1.42 ± 0.73	3.18 ± 0.62
	Peak systolic KE/CI, mJ/(l/min/m^2^)	0.37 ± 0.18	1.55 ± 0.38
	Peak diastolic KE/CI, mJ/(l/min/m^2^)	0.51 ± 0.15	1.93 ± 0.48
Right ventricle Fontan, *n* = 7 Controls, *n* = 8	EDVI, ml/m^2^	87 ± 20	96 ± 17
	ESVI, ml/m^2^	52 ± 13	40 ± 10
	EF, %	39 ± 7	59 ± 6
	CO, l/min	3.4 ± 2.0	6.8 ± 1.5
	CI, l/min/m^2^	2.9 ± 1.0	3.4 ± 0.5
	Peak systolic KE/SV, mJ/ml	0.043 ± 0.018	0.074 ± 0.014
	Peak diastolic KE/SV, mJ/ml	0.025 ± 0.012	0.034 ± 0.007
	Peak systolic KE/BSA, mJ/m^2^	0.78 ± 0.51	4.04 ± 0.87
	Peak diastolic KE/BSA, mJ/m^2^	0.96 ± 0.54	1.94 ± 0.70
	Peak systolic KE/CI, mJ/(l/min/m^2^)	0.79 ± 0.58	2.36 ± 0.65
	Peak diastolic KE/CI, mJ/(l/min/m^2^)	0.44 ± 0.31	1.11 ± 0.38

*RV* right ventricle; *LV* left ventricle; *EDVI* end-diastolic volume index; *ESVI* end-systolic volume index; *EF* ejection fraction; *CO* cardiac output; *CI* cardiac index; *KE* kinetic energy; *SV* stroke volume; *BSA* body surface area; *CI* cardiac index

No correlation was seen between ejection fraction and KE/SV regarding mean (*r*=−0.27; *p* = 0.40), peak systolic (*r*=−0.22; *p* = 0.50), and peak diastolic values (*r*=−0.13; *p* = 0.70). There were no correlations between peak systolic velocity and peak systolic KE (*r* = 0.36, *p* = 0.27) or peak diastolic velocity and peak diastolic KE (*r* = 0.11, *p* = 0.75).

There was a significant correlation between mean KE and ventricular volumes in both patients and controls. In patients, *r* = 0.9, *p* = 0.0008 for EDV and *r* = 0.9, *p* = 0.0004 for end-systolic volume (ESV); and in controls *r* = 0.7; *p* = 0.05 for EDV and *r* = 0.8; *p* = 0.02 for ESV.

**Fig. 4:**
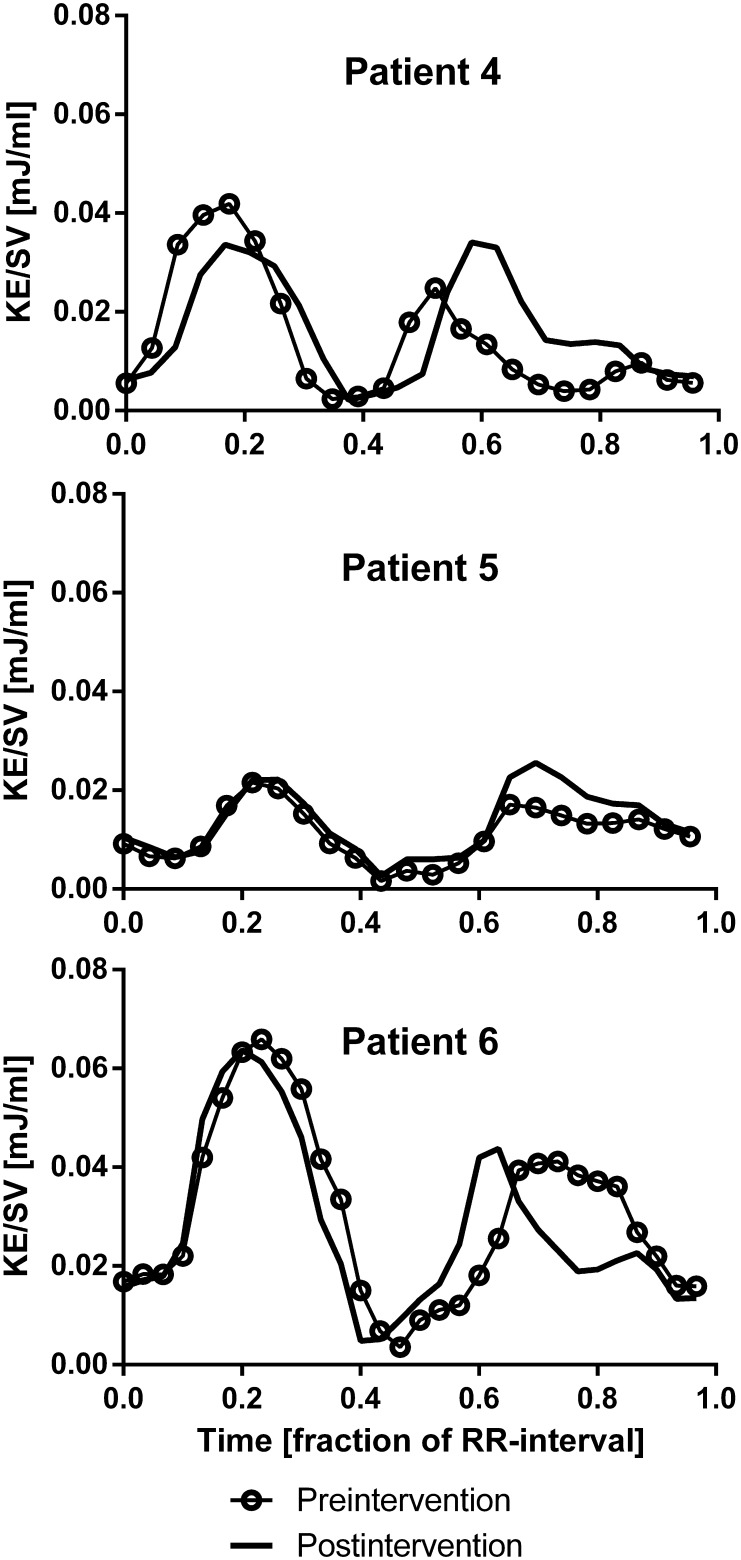
KE throughout the cardiac cycle in three patients before and after surgical or percutaneous intervention. Details of the intervention are listed in Table 2. Heart rate (beats/minute) pre-intervention/post-intervention was in patient 4: 103/87, patient 5: 85/83, patient 6: 63/80

The KE patterns in patients after intervention compared to baseline MRI are shown in Fig. 4. In all patients, the KE patterns were similar before and after intervention, but in patients 4 and 6 there was a change in the shape of the diastolic curve. Patient 4 had a surgical conversion of the extra cardiac conduit to a Y-graft from the hepatic veins to each pulmonary artery branch to better distribute the hepatic blood flow to the lungs [30, 31]. Before intervention, the curve had two diastolic peaks, but after surgery the early diastolic peak was higher and fused with the late diastolic peak. Patient 6 had a fused diastolic curve with a plateau before intervention; after coiling of the APC, the curve showed two diastolic peaks.

Interobserver variability for EDV was − 1 ± 12%.

### KE in the Fontan Pathway and Pulmonary Branches

KE in the tunnel or conduit from IVC could be quantified in all the patients. KE could not be analyzed in both PA branches in all patients and in one case in SVC due to stent artifacts or too short vessel. Mean KE in the tunnel/conduit (*n* = 11) was 0.0058 ± 0.0045 mJ/ml; in SVC (*n* = 10) 0.0093 ± 0.0023 mJ/ml; in left PA (*n* = 8) 0.018 ± 0.009; and in right PA (*n* = 8) 0.015 ± 0.006 mJ/ml. In 5/11 patients, KE could be quantified in SVC, the tunnel/conduit from the IVC, and both PA branches. In this subgroup, the mean KE was significantly higher in both PA branches together, 0.034 ± 0.006, than in the tunnel/conduit + SVC, 0.014 ± 0.006 (*p* = 0.008). The mean cross-sectional area of both PA branches together was 2.2 ± 1.2 cm^2^ and in the tunnel/conduit + SVC 4.4 ± 0.9 cm^2^.

## Discussion

This study shows that kinetic energy of blood in the single ventricle during the cardiac cycle in Fontan patients is dependent on the left or right morphology of the ventricle and that peak diastolic KE is lower in patients with Fontan circulation compared to controls.

Diastolic KE was larger than systolic KE in patients with LV morphology similar to the LV of healthy volunteers. In patients with an RV morphology, the KE pattern was similar to that of the RV in healthy volunteers with a higher systolic KE compared to diastolic KE. The mechanisms for these differences may be that during systole, blood flows with higher velocities within a longer right ventricular outflow tract compared to the shorter left ventricular outflow tract, resulting in higher KE within the heart on the right side compared to the left.

During diastole, the blood is accelerated into the left ventricle primarily by ventricular recoil leading to high KE. This is in contrast to the right ventricle where filling of the ventricle is mainly caused by movement of the atrioventricular plane toward the base of the heart over the blood in the atrium which requires less KE as the blood does not need to be accelerated into the ventricle(9). The interventricular difference of the LV as a pressure pump and the RV as a volume pump [15] is reflected in the findings that LV mass is an independent predictor of LV diastolic KE and RV end-diastolic volume an independent predictor of RV diastolic KE [19].

The study also shows that peak diastolic KE/SV is lower in patients with Fontan circulation compared to controls. These findings differ from results in adult heart failure patients [32] where diastolic KE indexed to stroke volume was increased compared to controls. The explanation for a lower KE in Fontan patients compared to patients with congestive heart failure may be that the impaired ventricular filling and the forward failure problem in Fontan patients result in intracardiac flow with lower velocities and that this is represented as a lower KE. This is in contrast to adult heart failure which is characterized by predominant backward failure and increased filling pressures and increased KE/SV, independent of etiology being ischemic or nonischemic cardiomyopathy. A contributing factor to the difference could be that 7 out of 11 patients had right ventricle morphology and thereby low peak diastolic KE, but the difference seems to remain when comparing Fontan patients with left ventricle morphology with left ventricles of controls, although the number of patients are low. The fact that the majority of Fontan patients had RV morphology also explains why there was no significant difference in peak KE/SV in systole when comparing the Fontan group with the LV of controls. However, the results indicate that the systolic KE will differ between patients and controls when ventricular morphology is taken into account. We did not find any difference in KE between stable patients and patients with complications, and this may also be explained by the larger effect of ventricular morphology on KE.

The high correlation between KE and ventricular volumes is explained by the fact that KE is calculated from both velocity and mass of the blood. The large differences in size and heart rates in the study population were the reason to index KE to stroke volume (SV). SV was chosen as more appropriate compared to ventricular volumes as EDV differs greatly with the type of heart defect in Fontan patients. Stroke volume is the net result of ventricular filling and contraction and as such intuitive for indexing. Another way to index could be to body surface area as in, for example, Jeong et al.´s article on patients with tetralogy of Fallot [23]. Kinetic energy could also be indexed to cardiac index, since this indexation has been used in other studies, but this will create a difference between pediatric and adult hearts as the cardiac index is similar but the mass of blood is much smaller in the small heart leading to lower KE. Therefore, we indexed to stroke volume to be able to compare small and large hearts.

Three patients in this study underwent a second examination after surgical or percutaneous intervention. The results show remarkably similar magnitude and basic pattern of KE over the cardiac cycle at the two examinations. This suggests that the KE pattern is quite specific to each patient. A possible explanation to the change in diastolic curve shape after intervention in patient 6 could be that the aortopulmonary collaterals before intervention caused an extra inflow with high pressure in the atrium leading to a plateau of the KE curve during diastole. This explanation is supported by the fact that all patients in this study who had clinically significant aortopulmonary collaterals (APCs) also had a plateau in the diastolic part of the KE curve. The rise in diastolic KE in patient 4 after surgery might reflect an increase in pulmonary arteriovenous fistulas coherent with the clinical picture. These results address the complexity in evaluating KE in patients with Fontan circulation and indicate that the clinical implication for using KE in this patient group primarily is to better understand the physiology and to follow the patients on an individual basis. In our study, only one patient had fenestration between the conduit and the atria. The possible effects on ventricular KE from flow through the fenestration are not known but it may be similar to APC with a diastolic plateau and higher KE in diastole if the fenestration causes higher preload. In one of the patients in whom no significant APC could be identified, there was a diastolic plateau. Larger numbers of Fontan patients with KE are needed to see if the diastolic plateau pattern is related to other clinical parameters than APC flow. Recently, Schiavazzi et al. used modeling to calculate the effects of left pulmonary artery stenosis on hemodynamics in single ventricles stage II surgery and found that the stenosis needed to be quite severe, >65% of the diameter, to be hemodynamically significant [33]. This is in line with the findings of unchanged ventricular KE pattern before and after stenting of a left pulmonary stenosis in one of our study patients.

The analysis of KE indexed for stroke volume in the Fontan pathway showed a significant increase in KE from the caval veins to the PA branches. This is probably explained by the smaller cross-sectional area of the PA branches compared to the caval veins.

Another way to analyze KE in the ventricle is to divide it based on whether the volume goes directly from the atria to the aorta in the same heart beat or if it stays one or more heart beats in the ventricle [34]. Eriksson et al. [21] have suggested that KE might reflect ventricular dysfunction at an early stage. They found altered diastolic flow routes and impaired preservation of KE in late diastole in the LV of patients with dilated cardiomyopathy compared with healthy subjects. The changes in flow route and energetics were seen despite clinical compensation and might be useful as subclinical markers of LV dysfunction. Future studies using this analysis will provide information if this will give additional insights into the Fontan physiology. 4D flow has also been used to quantify kinetic energy of turbulent flow and this energy may be lost to heat [20]. In Fontan patients with slower velocities, turbulent flow is less likely and we did not find any patients with signal drop in the magnitude images which would have indicated turbulent flow.

A study of kinetic energy in patients with Tetralogy of Fallot, recently published by Jeong et al [23], shows that patients had a tendency to higher systolic peak KE compared to healthy volunteers and that a change in ventricular KE may prove to be an indication of ventricular dysfunction. Together with these studies and the results from studies in adult patients with heart failure [32], this study indicates that KE might be a useful noninvasive method of monitoring patients and detect signs of ventricular and circulatory dysfunction [35]. The finding that there is no correlation between KE and peak velocity shows that quantifying the entire velocity field in three dimensions of the entire ventricle has additional information compared to peak velocities. Kinetic energy as a single measure will probably not predict subsequent ventricular function but the future use will be in the context of several hemodynamic measures.

In Fontan patients, breathing contributes to the return of blood to the lungs to a higher extent than in normal physiology [36]. Therefore, we acquired flow measurements during free breathing and not respiratory triggered to end-inspiration or end-expiration, to be able to get mean flow throughout the respiratory cycle.

## Study Limitations

The study population is small and therefore statistical comparisons of the subgroups of left and right ventricular morphology and patients before and after intervention could not be made. Conclusions of the effect on KE of APC’s or interventions are also limited by the small sample size, but the data presented can point to applications where future studies may provide insights of Fontan physiology. In the Fontan group, five out of eleven patients were ventilated during the examination and this may affect the results. However, the KE results from the ventilated patients do not appear different from the nonventilated patients, but this needs to be further investigated. The patient who was under anesthesia pre-intervention (*n* = 1) was also under anesthesia at the post-intervention CMR. Heart rates varied between examinations, but indexed KE remained similar independent of heart rate. There may be age-related differences in KE and in this study we did not have age matched controls. However, this was compensated for by indexing KE to SV.

## Conclusion

This is to our knowledge the first study that quantifies the intraventricular KE of Fontan patients. KE might be a useful noninvasive method of monitoring Fontan patients and detect signs of ventricular and circulatory dysfunction. Further studies are needed to determine if changes in ventricular KE can provide early information on ventricular dysfunction or for example the significance of aortopulmonary collaterals and guide medical and surgical intervention.

## Electronic Supplementary Material

Below is the link to the electronic supplementary material.

Movie 1. Movie showing how image acquisition and analysis is done to obtain ventricular kinetic energy using 4D flow MRI. (MP4 23379 KB)

Movie 2. Movie showing an example of intracardiac and cavopulmonary blood flow in a patient with Fontan circulation. (MP4 4104 KB)

## Abbreviations

APC: Aortopulmonary collaterals
ASD: Atrial septal defect
AVSD: Atrioventricular septal defect
BSA: Body surface area
CI: Cardiac index
CMR: Cardiac magnetic resonance
CO: Cardiac output
CoA: Coarctation of the aorta
DILV: Double inlet left ventricle
DORV: Double outlet right ventricle
EDVI: End-diastolic volume index
EF: Ejection fraction
ESVI: End-systolic volume index
HLHS: Hypoplastic left heart syndrome
IVC: Inferior vena cava
KE: Kinetic energy
LPA: Left pulmonary artery
LV: Left ventricle
MAPCA: Major aortopulmonary collateral arteries
NYHA: New York Heart Association Functional Classification
PLE: Protein-losing enteropathy
PS: Pulmonary stenosis
RV: Right ventricle
SV: Stroke volume
SVC: Superior vena cava
TAPVD: Total anomalous pulmonary venous drainage
TGA: Transposition of the great arteries
VSD: Ventricular septal defect
4D PC-MRI: Four-dimensional phase-contrast magnetic resonance imaging
